# Medial fascia lata perforator flap: Anatomical basis and clinical application as a pedicled or free flap

**DOI:** 10.1016/j.jpra.2023.07.009

**Published:** 2023-08-04

**Authors:** Gabriele Delia, Fabiana Battaglia, Emanuele Cigna, Leonardo Ioppolo, Michele Maruccia, Francesco Stagno d'Alcontres

**Affiliations:** a Plastic Reconstructive and Aesthetic Surgery Unit, University of Messina, Policlinico 'G. Martino', Via Consolare Valeria 1, 98125 Messina, Italy; b Plastic Surgery and Microsurgery Unit, Department of Translational Research and New Technologies in Medicine and Surgery, University of Pisa, 56122 Pisa, Italy; c Marrelli Hospital, Crotone, Italy; d Section of Plastic and Reconstructive Surgery, Department of Emergency and Organ Transplantation, University of Bari, Bari, Italy

**Keywords:** Adipofascial flaps, Gracilis flap, TUG flap, Cadaveric study, Anatomical dissections, Femoralis fascia, Fascia lata flap

## Abstract

Adipofascial flaps have proven to be an excellent tool for multipurpose reconstruction as free or pedicled flaps. The anatomical studies in this field are now focused on improving esthetics in reconstruction while maintaining a minimal donor site morbidity. An anatomical cadaver study has been carried out to investigate the medial thigh region as a potential donor site for adipofascial flaps.

Eighteen thighs from fresh cadavers were dissected and a new territory with autonomous vascular supply was defined through vascular injection, anatomical dissection, transillumination, and angiography. Cutaneous access was made in a “T” shape. The fascia harvests had to be centered on the adductor longus and gracilis muscles bearing in mind the position of the flap pedicle. The fascial flap was isolated from adductor longus and gracilis muscles and isolated on his pedicle (medial circumflex femoral artery).

After our anatomical study, we used the flap in 2 clinical cases. The results of our anatomical study and clinical cases confirmed the suitability and reliability of a new flap: the “Medial Fascia Lata Flap.” Flap size ranged from 20 to 25 cm and has the advantage of preserving the functionality of the thigh muscles. The study showed that the “Medial Fascia Lata Flap” is easy to harvest, and the resulting scar is concealed. In consideration of its suitability, reliability and aesthetical advantages, it could be proposed as a good option in selected cases.

## Introduction

In reconstructive surgery, the medial thigh region has long been considered a flaps’ good source. The gracilis muscle is widely applied for different reconstructive options in plastic surgery, orthopedic, head, and neck surgery. It provides an acceptable donor site mobility and the adductor function is preserved. The fascia lata receives sufficient blood supply via the prefascial and subfascial plexus. These attributes make this flap a good alternative to other flaps for reconstruction. However, the resulting donor site scar and the length of the vascular pedicle, limited to 6 to 10 cm, need to be considered.^1^

It is also well known as an extremely versatile flap, adapting brilliantly to different anatomical situations^2^, even in case of visible scars and limited functions, such as in the transverse upper gracilis (TUG) flap.^3^

In the early 90’s, interest in adipofascial flaps grows and they have already been in the plastic surgeon's armamentarium.^4^ It's low donor site morbidity, minimal tissue defect, and scar make it an ideal donor site. However, after widespread application of these flaps, the description and clinical applications of perforator flaps have reduced their use, nevertheless, in some cases, they are still irreplaceable.

This article presents an anatomical study of the medial femoral region on fresh cadavers in order to isolate a new flap. The medial Fascia Lata Perforator Flap (MFLPF).

## Materials and methods

In the Anatomy Laboratory of Bordeaux 2 University (France) 18 fresh thighs from cadavers were studied. There were 11 males and 7 females, 8 from the left and 10 from the right lower limbs. After an injection of a white Micropaque® solution in the profunda femoris and in the medial circumflex femoral arteries, the femoralis fascia was harvested upon loupe magnification (4 ×) as a pedicled flap and then as a free flap. Transillumination as well as standard X-rays were used to highlight the vascular arborisation*.* The Shinwa Gauge instrument is used for the vessels measurements.

### Flap dissection

Flap dissection was made by placing the cadavers in a supine position with the limb in abduction and the knee and hip in flexion. The surgeon's position in the middle of the thighs. Palpable landmarks were fixed at the adductor longus muscle (both insertions and muscle belly), the pubic tubercle, and the medial tibial condyle.

With the thigh abducted, the first muscle palpable on its medial surface is the adductor longus, and the proximal insertion of gracilis is located just behind. Its muscular fibers run from the pubic tubercle to the medial tibial condyle, and its vascular pedicle is located around 10 cm under the pubic tubercle.

**Figure 1:**
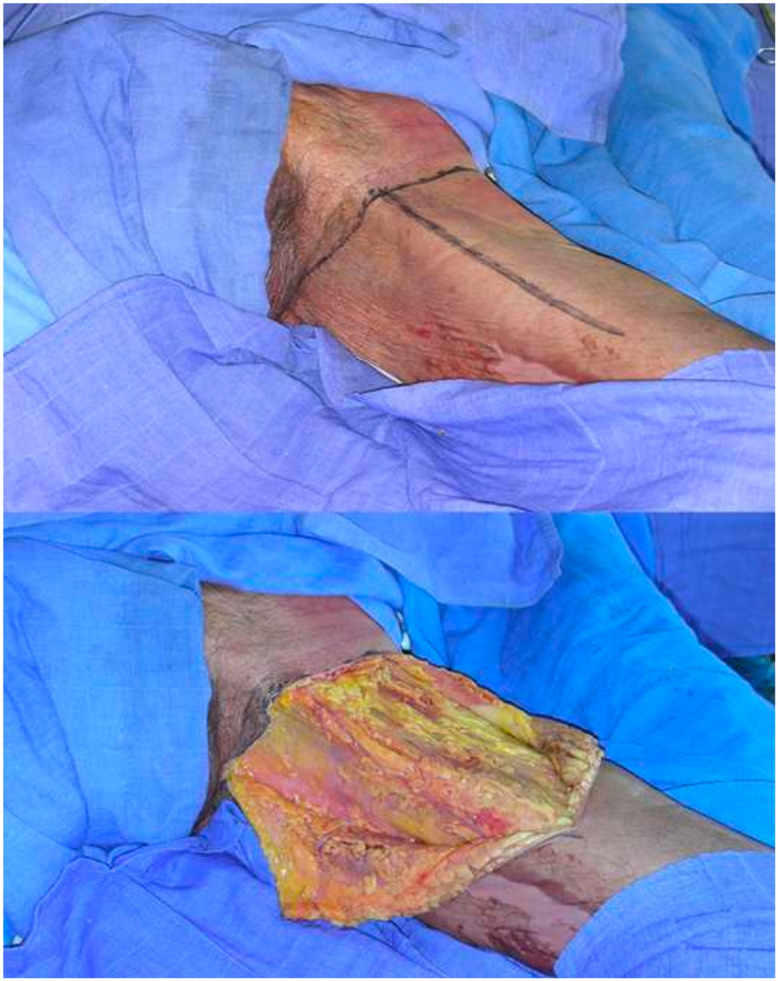
Anatomical landmarks are pointed out: the adductor longus muscle (both insertions and muscle belly), the pubic tubercle and the medial tibial condyle. A “T” shaped incision is planned. The vertical component of the T corresponds to the cutaneous projection of the adductor longus muscle, as well as the horizontal component, which is located parallel to the groin and 2-3 cm lower. Skin flaps including superficial fat are elevated, leaving the deep fat attached to the femoral fascia.

Cutaneous access was marked in a “T” shape. The vertical component of the T corresponded to the cutaneous projection of the adductor longus muscle, while the horizontal component was located parallel to the thigh's root and 2 to 3 cm lower. Bookpage skin flaps were elevated just over the femoral fascia, together with a thin layer of superficial fat, taking care to leave the deep fat attached to the underlying fascia. The fascia harvests had to be centered on the adductor longus and gracilis muscles bearing in mind the position of the flap pedicle (Figure 1).

The fascia was first incised on the lateral side and dissected up to the adductor longus muscle. Adductor longus muscle aponeurosis was then incised, and the medial portion of the muscle was freed from its fascial envelope. The descending branch of the medial circumflex femoral artery, located 8 to 10 cm under the pubic tubercle, was then exposed.

**Figure 2:**
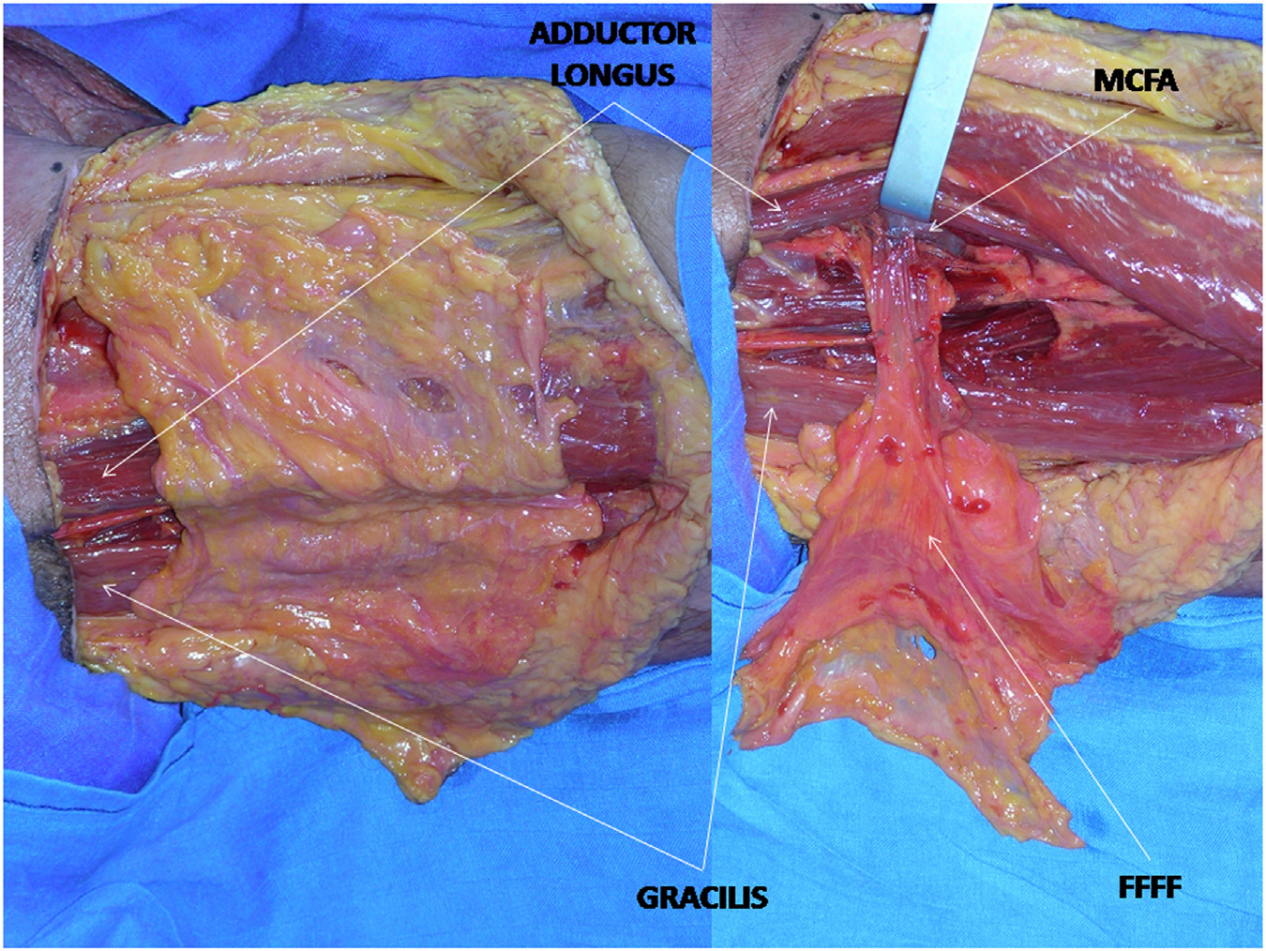
The fascia is first incised on the lateral side and dissected up to the adductor longus muscle. Adductor longus muscle aponeurosis is then incised and the medial portion of the adductor longus is freed from its fascial envelope. Fascia harvesting must be centered on the adductor longus and gracilis muscles. The descending branch of the medial circumflex femoral artery, located 8-10 cm under the pubic tubercle, is then exposed. Fascial dissection is then continued on the medial side cutting and freeing the gracilis aponeurosis, allowing complete exposure of the muscle belly.

Fascial dissection was then continued on the medial side and back to front, cutting and freeing the gracilis aponeurosis that allows complete exposure of the muscle belly (Figure 2).

**Figure 3:**
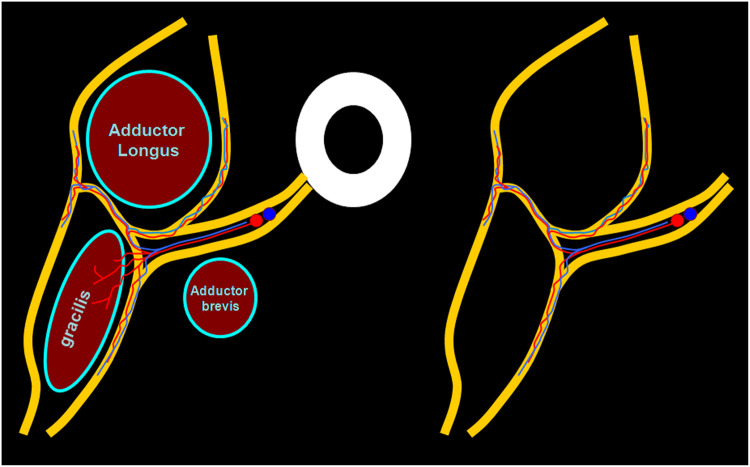
Diagram of the flap vascularization.

The flap was then isolated on its pedicle, and the length of its dissection continued according to the reconstructive requirements. A diagram of the flap vascularization is illustrated (Figure 3).

The pedicle is then isolated to its source on the deep femoral. The fascial flap's sizes can be adapted to the reconstructive needs. The average width is 20.38 cm, while the average length is about 22.66 cm, with a slight difference between men and women: the average width in men is 20.54 cm, whereas in women it is 20.14 cm; the average length in men is about 23.72 cm, whereas in women it is 21 cm (Table 1).

**Table 1:** N number of dissection. Sex of lower limb cadavers. Side of lower limb cadavers. BFFF branches for femoralis fascia. BFGM branches for gracilis muscle. Flap Size Harvested

N	SEX	SIDE	BFFF	BFGM	FSH
1	M	L	4	3	20 x 25
2	M	L	3	3	20 x 22
3	F	R	4	2	16 x 20
4	M	L	3	4	21 x 25
5	F	R	4	3	20 x 21
6	F	R	4	2	24 x 24
7	M	R	3	3	21 x 24
8	M	R	3	3	22 x 24
9	F	R	3	3	19 x 20
10	M	L	4	3	20 x 22
11	F	L	4	3	22 x 18
12	F	R	2	3	19 x 21
13	F	L	3	3	21 x 23
14	M	R	3	3	19 x 23
15	M	L	4	3	22 x 25
16	M	L	2	2	20 x 24
17	M	R	4	2	21 x 24
18	M	R	3	3	20 x 23

20,38 Total average width

22,66 Total average lenght

20,54 Average width for males

23,72 Average lenght for males

20,14 Average width for males

21 Average lenght for males

**Figure 4:**
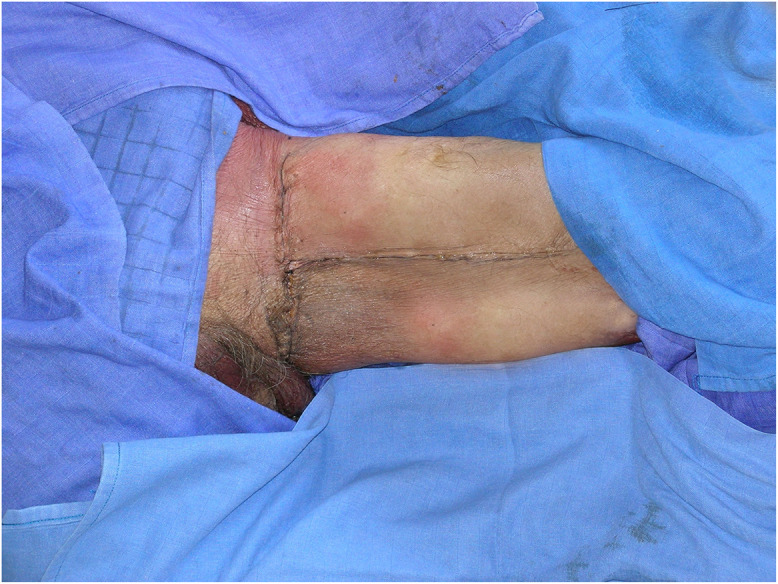
The donor site after closure: the scar falls in a perfectly hidden area.

After fascial flap elevation, the donor site was closed in a T-Y shape, producing a hidden scar on the inner thigh surface (Figure 4) like the thigh plasty.

Flap's dressing is not required even if they're hidden, in the opposite they can be daily dressed with fat gauze or grafted immediately.

### Transillumination

**Figure 5:**
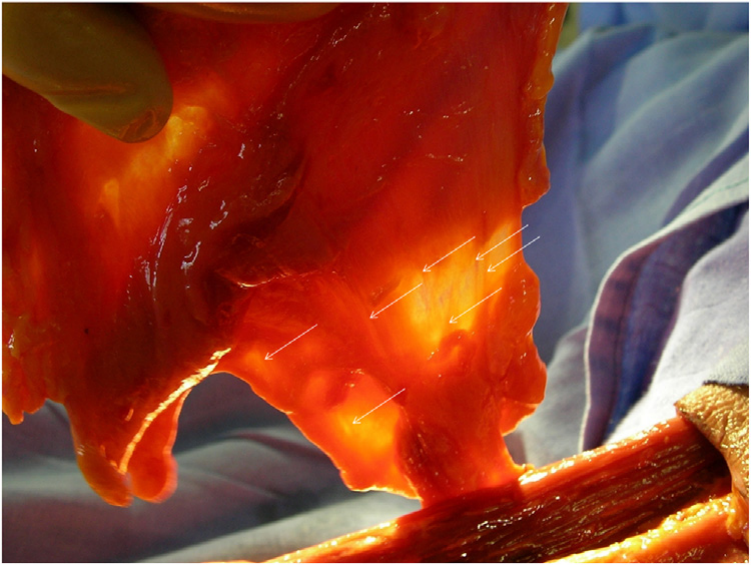
Transillumination demonstrating the vascular supply of fascial flap: the medial circumflex femoral artery enters the fascial tissue spreading into several vascular branches.

Transillumination was used to examine all 18 flaps before pedicle division. All 18 flaps were examined by transillumination before pedicle division. The vascular arborisation was easily visualized, showing a rich vascular tree of the flap (Figure 5).

### Radioangiography

**Figure 6:**
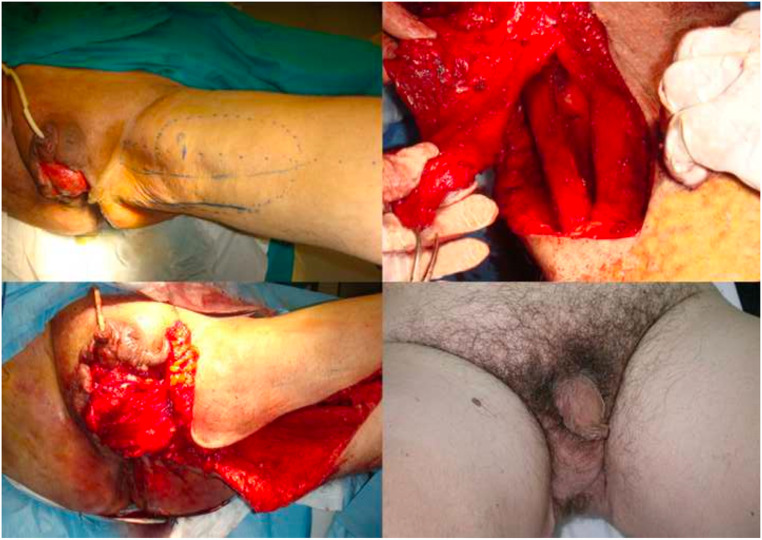
Radioangiographic specimen showing the medial circumflex femoral artery proving several branches for the femoral fascia in the territory of adductor longus. At least 3 of these branches have a significant caliber, they run through the femoral fascia, they give several fascial branches and they finally enter the gracilis belly.

After the flap harvest and pedicle division, the artery was perfused with a heparinated solution to wash the vascular network and visualize any remaining leaks from the flap or pedicle, which were ligated. The femoralis profunda artery was then slowly injected with 50 cc of Micropaque® solution. After the injection, each flap was frozen at −4°C for 24 hours and radiograms were performed the day after (Figure 6).

## Results

### Dissection

The fascia was entirely freed from its surrounding structures and remained attached only to the gracilis pedicle. The vascular pedicle could be traced from the profunda femoris to increase its length.

The dissection left the gracilis muscle on its site, while its vascular supply, which came from its secondary pedicles located 8 to 10 cm distally, together with the preservation of the motor nerve.

The adipofascial paddle flap could be harvested up to 30 × 20 cm.

At the end of surgical dissection, gracilis contractile activities were performed by electrostimulation of the motor nerve.

### Microanatomy of Medial Circumflex Femoral Artery (MCFA): vascular injection

The dissections of the medial circumflex femoral artery (MCFA) showed several branches coming off the femoral fascia in the territory of the adductor longus. At least 3 of these branches were of a significant caliber (about 1 mm diameter), running through the medial fascia lata. They generate several fascial branches before entering the gracilis muscle belly. The femoral fascia could then be pedicled on the medial circumflex femoral vessels. The fascial pedicle had been visualized outside the muscle, no intramuscular dissection was needed, muscular vessels were then ligated and sectioned.

In our anatomical study MCFA provides an average of 3 to 5 major branches to the femoral fascia.

### Transillumination

Transillumination was used to examine all eishowed the vascular supply of the fascia: the MCFA entered the fascial tissue spreading into several vascular branches and branchlets (Figure 5).

### Flap Angiogram

All angiograms showed an extremely rich fascial blood supply coming from the MCFA. This artery provided several branches, supplying the whole flap area (Figure 6).

## Discussion

A number of free fascia transfers, such as temporoparietal fascial flap, serratus anterior fascial flap, scapular adipofascial flap, thoracodorsal fascial flap, have proven to have distinct advantages over muscle, myocutaneous, and skin flap. They offer the advantages of a thin pliable vascularized tissue without bulk, rapid healing, high resistance to infection, decreased scarring and adhesions, improved tendon gliding and excursions, and conforming to any three-dimensional cavities or surfaces.^5^^,^^6^ Their clinical use for noble tissue covering, such as nerves, vessels, tendons and bones, as well as for reconstruction in case of poor blood supply of the recipient bed and in scarred or irradiated skin, has been successfully proposed. They are usually harvested with a negligible morbidity of the donor site.

The fascial flap must include, together with the fascia, a thin layer of subcutaneous tissue to protect the vascular network situated immediately above the fascia. However, after the widespread clinical application of perforator free or pedicled flaps, the use of the adipofascial flaps has dramatically fallen. Additionally, donor site mobility is reduced in perforator flaps. At the same time, skin coverage is provided by giving all reconstructive needs without requiring a second debunking procedure after reconstruction.

Adipofascial flaps are today mainly indicated for large tissue defects such as scalp or lower limb defects. They are also used in cases of gliding surface reconstruction for the upper limb^7^^,^^8^ and for the planning of prefabricated flaps.^9^, ^10^, ^11^ Among them the greater omentum free flap has maintained the major role in the possibility of very large tissue coverage. Although it can be endoscopically elevated^12^, thus reducing its donor site morbidity, due to its well-known drawbacks^8^, its application is gradually decreasing and being replaced, by other flaps, in primis perforator flaps*.*^13^

Among adipofascial flaps, the anterolateral thigh flap, being the largest one, has become a strong point and used for this indication.^12^^,^^14^^,^^15^ In contrast, as an adipofascial flap, the anterolateral thigh free flap has 2 main disadvantages: the elevation of the perforator muscle with the related tedious dissection to supply the flap, and the possible wound bed infection.

The femoral fascia, also called fascia lata, represents a large source of fascial and subcutaneous tissue surrounding the whole thigh, from the antero-lateral to the posterior region.^16^

A number of studies, described medial thigh vascular supply have been presented for coverage of wounds at this region. It has proven to be highly vascularized and a rich source of flaps with a limited donor site morbidity.

Hallock^17^ showed that 1 to 4 significant musculocutaneous perforators can be found emanating through the proximal gracilis muscle, within a region approximately 3 cm in radius centered over its vascular hilum.

Interestingly, Whetzel and Lechtman^1^, observed that the blood supply of thigh skin, located over the gracilis muscle, comes primarily from vessels traveling around, rather than through, the gracilis muscle. These vessels run through the perigracilis fascial tissue and the inclusion of this fascia, while harvesting a gracilis myofasciocutaneous flap, should be recommended to enhance its blood supply. Based on anatomical study, skin perfusion of the medial thigh mainly occurs via communications between the proximal main pedicle of the medial femoral circumflex pedicle and the fascial vascular network of the superficial femoral septocutaneous vessels, rather than through direct musculocutaneous gracilis perforators.

Later on, Zheng et al.^18^ showed that even the muscular branches of the medial vastus muscle give out constant perforating branches.

Wei-Ren and Taylor^19^ reported the angiosomes of the thigh, showing how the branches of the profunda artery provide septocutaneous perforators. They pierce the deep fascia in vertical rows between the semitendinosus and semimembranosus muscles medially and between the biceps femoris and iliotibial tract laterally. Moreover, musculocutaneous perforators emerged from the vastus lateralis muscle.

In their study, Peek et al.^20^ showed that perforators were either penetrating the gracilis muscle (musculocutaneous) or running in the septum between the adductor longus and gracilis muscles (septocutaneous).

Lastly, Eom et al.^21^ demonstrated that approximately 90 percent of the perforators originate from the MCFA, one-third are the musculocutaneous type and two-thirds are the septocutaneous type. The remaining 10 percent were from multiple sources: from the deep femoral artery and from a septocutaneous perforator between the semitendinosus and adductor muscles. They used a suprafascial approach to raise flaps having a transverse length up to 15 cm, reaching the femoral vessels. With additional debulking of the flap, the circulation was not compromised. Other studies emphasize that the subfascial approach should be used to ensure a safe vascular supply. Our findings fit perfectly with previous studies, taking care to leave the deep fat attached to the underlying fascia, as Kappler et al.^22^ and Lykoudis et al.^23^ stated in their reports.

The vascular plexus of the fascia is the basis for the medial thigh flaps, regardless of whether the blood supply is from the posterior vessels, the gracilis pedicle perforators, the external pudendal vessels, or other branches of the superficial femoral vessels. As shown in this study, the MCFA gives off several branches for the femoral fascia in the territory of the adductor longus before entering the gracilis belly. The dissections of the MCFA showed several terminal branches of significant caliber (>0.5 mm) in the adductors territory, running through the femoral fascia, generating some fascial branches before entering the gracilis muscle belly.

Advantages: these flaps have a perfect adaptability to loss of substance without bulking effect, filling of cavities, excellent vascularization, they can also be buried under the skin.

Disadvantages: frequent dressings, during the first days may tend to dry out, they need a skin graft one they have adapted to the bottom of the lesion in the case they are used unburied.

## Clinical Cases

The reliability of the study has been confirmed by a clinical application in which a femoral fascia pedicled flap, based on the MCFA, has been successfully applied.

**Figure 7:**
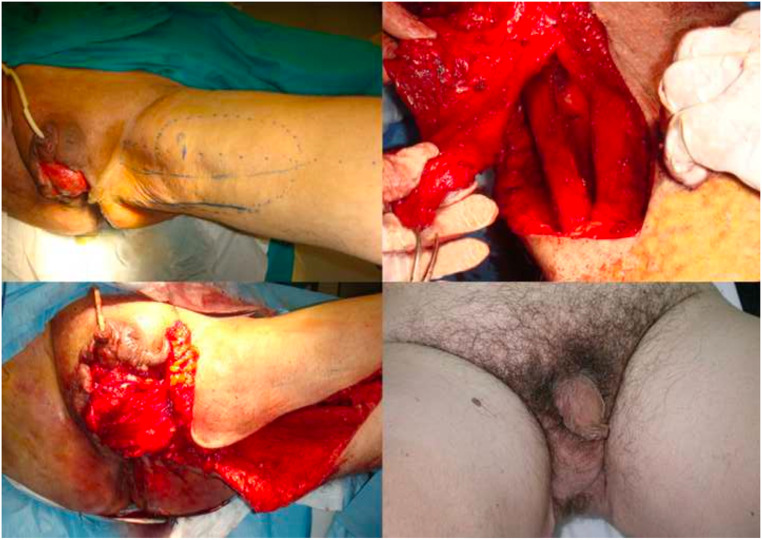
Fournier's gangrene complication with loss of scrotal substance. It is covered with perforating flap of medial pedicled fascia lata.

The adipofascial flap was used as a pedicled flap in a patient suffering from Fournier's gangrene outcomes. In this case, after surgical debridement and application of Vac Therapy for 5 days, the adipofascial flap was harvested to cover the scrotal skin defect (Figure 7).

The MFLPF flap was also used as a free flap in 2 cases of limb reconstruction.

**Figure 8:**
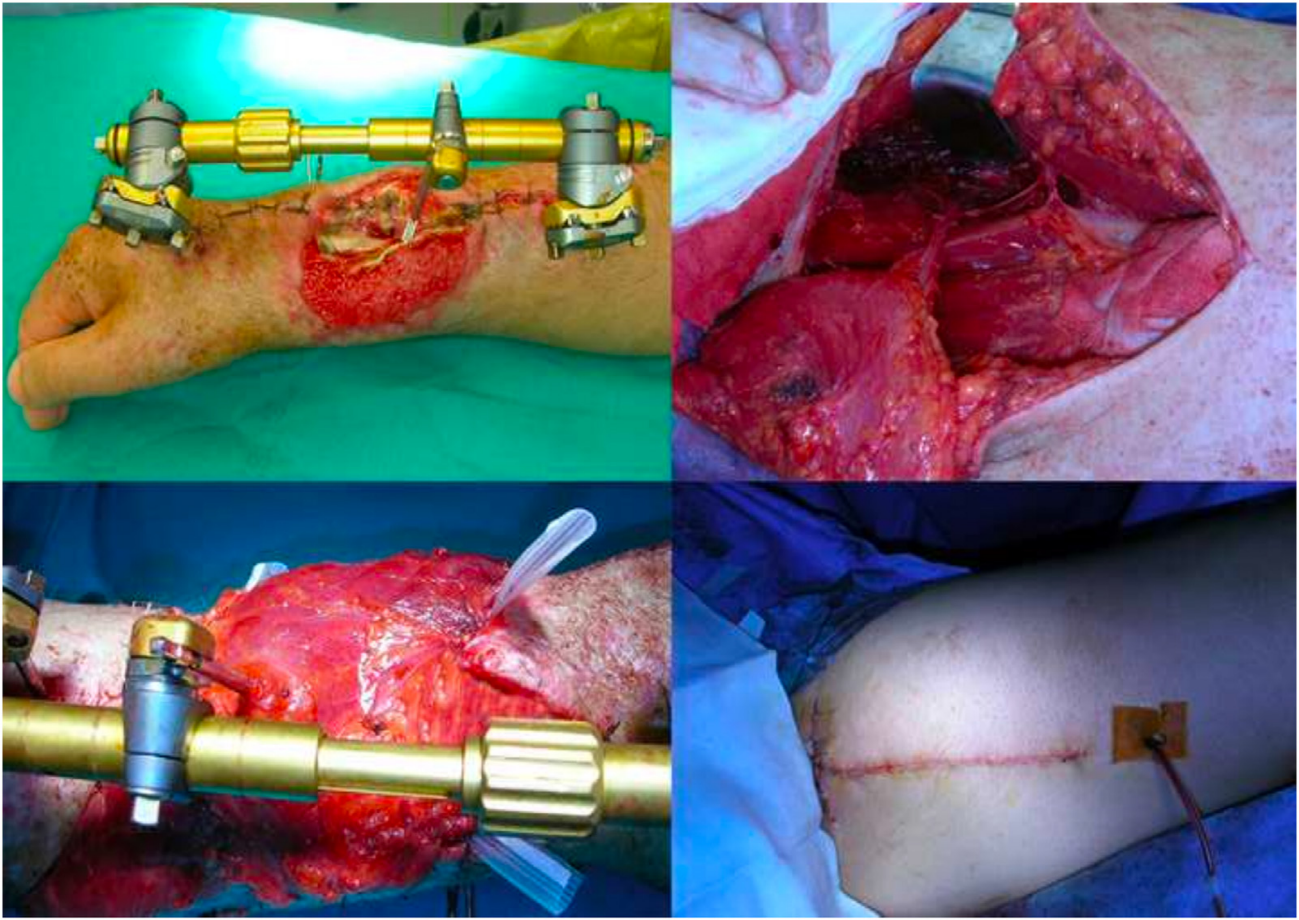
Right forearm loss of substance with osteotendinous exposure. It is covered with free flap of medial fascia lata.

**Figure 9:**
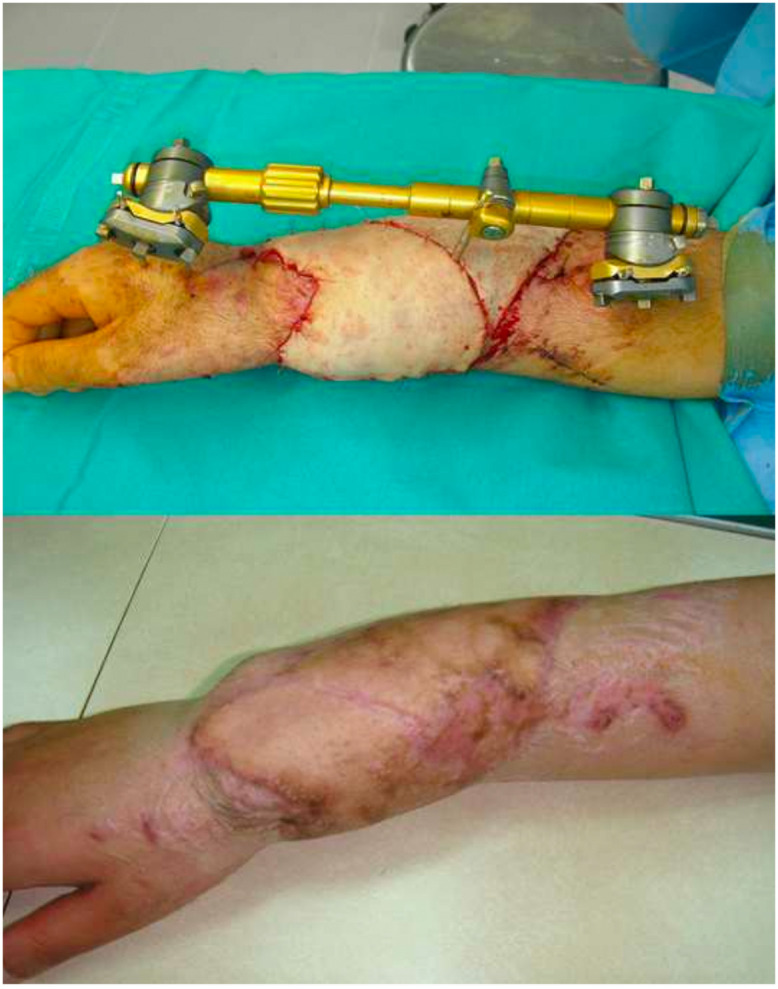
Postoperative result after skin grafting and result after 1 year.

A patient suffering from complex trauma of the superior right limb, with exposed fracture, was treated with a microvascular MFLPF, and then covered with split thickness skin grafts (Figs. 8, 9).

## Conclusion

Adipofascial flaps have been valuable for complex plastic surgery reconstruction, but in the past, adipofascial flaps were commonly used in complex plastic surgery. Now, perforator flaps have taken the lead, thanks to their one-stage reconstruction, reduced donor site morbidity, and excellent results for small-medium size reconstructions.

The femoralis fascia flap, supported by this study, offers a highly vascularized alternative. It is versatile, thin, and ideal for various reconstructions from scalp to limbs, even as a scaffold for prefabricated flaps. Plus, it preserves gracilis muscle function and conceals scars with minimal donor site issues. Embracing this flap could pave the way for groundbreaking outcomes.
